# Re-biopsy in lupus nephritis: Re-biópsia em nefrite lúpica

**DOI:** 10.1590/1516-3180.2014.9521711

**Published:** 2014-11-28

**Authors:** Raida Husein, Beatriz Camargo de Souza, Thanis Meyer, Thelma Larocca Skare

**Affiliations:** I MD. Rheumatology Fellow, Hospital Universitário Evangélico de Curitiba, Paraná, Brazil.; II Student, School of Medicine, Faculdade Evangélica do Paraná (Fepar), Curitiba, Paraná, Brazil.; III MD, PhD. Head of Rheumatology Unit, Hospital Universitário Evangélico de Curitiba, Paraná, Brazil.

## TO THE EDITOR

Systemic lupus erythematosus (SLE) is a heterogeneous disease and its clinical profile may be influenced by the patient’s genetic background.^1^^,^^2^ Lupus nephritis is a common manifestation appearing in up to 50% of the cases and it has an important impact on morbidity and mortality.^3^ Studies have shown evidence for linkage between lupus nephritis and the 1q 41-42 chromosome region.^1^ Lupus nephritis is considered to be more common and more severe in African-Americans and Hispanics.^1^ In this context, it is reasonable to believe that the impact of lupus nephritis varies according to the population, thus highlighting the need for local studies.

When a systemic lupus erythematosus patient has nephritis, there is a 20-30% probability of a new kidney flare per patient-year of follow-up.^3^ Many of these are mild, but repeated flares may end up in renal failure.^3^ The classes of lupus nephritis^4^ guide the treatment and prognosis and may change from one to another during a disease flare-up.^2^^,^^4^ Some authors have advised repeating biopsies during a lupus nephritis flare-up in order to determine the most effective treatment;^2^ others have suggested that the original classification determines the need for a repeated biopsy.^4^ An analysis on 35 patients by Daleboudt et al.^4^ showed that patients with proliferative lesions rarely switch to pure nonproliferative nephritis. Since the treatment for the proliferative classes (3 and 4) is the same, these authors considered that repeating the biopsy was unnecessary. Another study, on 156 Chinese lupus nephritis patients,^2^ showed that changes were common and that the histological class could not be predicted from the baseline clinical or biochemical parameters. These authors stressed the need for a second biopsy.

To better understand the behavior of the histological pattern of lupus nephritis in disease flares in our population, we retrospectively studied all the systemic lupus erythematosus patients with renal involvement who attended our clinic over the past 12 months. This rheumatology clinic belongs to a university hospital that attended 238 systemic lupus erythematosus patients within the Brazilian National Health System over the past year. By means of biopsies, we identified renal involvement in 98/238 patients (41.1%): 10 males and 88 females; median age 37.5 years; and median disease duration 9.6 years. In the first flare, 12.2% presented class 2; 21.4%, class 3; 41.8%, class 4; 18.3%, class 5; 3%, class 6; and 3%, class 3+5. In 22/98 (22.4%), there was a second biopsy-proven renal flare, which occurred after a mean time of 3.7 ± 2.2 years. Patients with a second renal biopsy due to other indications were not considered. The results are compared in Table 1. This table shows that none of the class 2 patients, 33% of class 3, 11% of class 4 and 75% of class 5 remained in the same class. In terms of proliferative classes (n = 15) and nonproliferative classes (n = 7), we observed a change in 5/15 (33%) from proliferative to nonproliferative and a change in 3/7 (42%) from nonproliferative to proliferative.

**Table 1.**
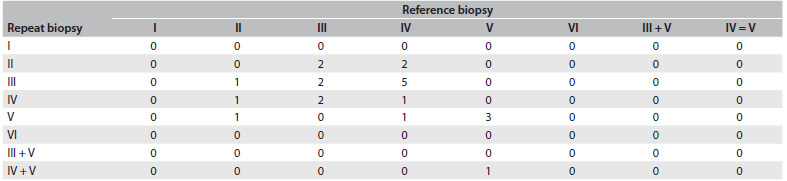
Change in ISN/RPS (International Society of Nephrology and Renal Pathology Society) biopsy classification^3^ for 22 lupus nephritis patients

Glomerulonephritis classes have prognostic implication and the most important reason for performing a new biopsy is to classify such cases correctly in order to decide on the treatment.^5^ Transitions between proliferative classes (e.g. class III to class IV and vice versa) do not impact on the prognosis or on selecting the therapy, since both of these are guided by the proliferative component of the lesion.^4^ Nevertheless, the switch from proliferative to non-proliferative lesions and vice versa, as was seen in a good percentage of our sample, will have clear consequences with regard to the treatment, so as to avoid undertreatment in a new class III or IV case, or unnecessarily increased immunosuppression in a class V case.^4^ So far, no good studies on the value of re-biopsy on long-term disease prognosis have yet been conducted.

We conclude that changes to the histological classification of lupus nephritis in the population of systemic lupus erythematosus patients studied here were common. In the present sample, Class V was the class with greatest constancy. Until more data is available on our population, it is advisable to perform a second renal biopsy in the case of a new flare-up of lupus nephritis.
